# Voluntary Exercise Positively Affects the Recovery of Long-Nerve Gap Injury Following Tube-Bridging with Human Skeletal Muscle-Derived Stem Cell Transplantation

**DOI:** 10.3390/jcm7040067

**Published:** 2018-04-02

**Authors:** Hiroya Seta, Daisuke Maki, Akihito Kazuno, Ippei Yamato, Nobuyuki Nakajima, Shuichi Soeda, Yoshiyasu Uchiyama, Tetsuro Tamaki

**Affiliations:** 1 Muscle Physiology & Cell Biology Unit, Tokai University School of Medicine, 143 Shimokasuya, Isehara, Kanagawa 259-1193, Japan; hrystx0168@gmail.com (H.S.); d.maki@tokai.ac.jp (D.M.); tokaikazuno@yahoo.co.jp (A.K.); ippei-y@is.icc.u-tokai.ac.jp (I.Y.); nakaji.n@is.icc.u-tokai.ac.jp (N.N.); shu10206@kaname-clinic.com (S.S.); y-uchi@is.icc.u-tokai.ac.jp (Y.U.); 2 Department of Human Structure and Function, Tokai University School of Medicine, 143 Shimokasuya, Isehara, Kanagawa 259-1193, Japan; 3 Department of Otolaryngology, Tokai University School of Medicine, 143 Shimokasuya, Isehara, Kanagawa 259-1193, Japan; 4 Department of Surgery, Tokai University School of Medicine, 143 Shimokasuya, Isehara, Kanagawa 259-1193, Japan; 5 Department of Medical Education, Tokai University School of Medicine, 143 Shimokasuya, Isehara, Kanagawa 259-1193, Japan; 6 Department of Urology, Tokai University School of Medicine, 143 Shimokasuya, Isehara, Kanagawa 259-1193, Japan; 7 Department of Orthopedics, Tokai University School of Medicine, 143 Shimokasuya, Isehara, Kanagawa 259-1193, Japan

**Keywords:** motor nerve function, tetanic tension, sensory nerve function, p75, N200, myelin basic protein

## Abstract

The therapeutic effects of voluntary exercise on the recovery of long-gap nerve injury following the bridging of an acellular conduit filled with human skeletal muscle-derived stem cells (Sk-SCs) have been described. Human Sk-SCs were sorted as CD34^+^/45^−^ (Sk-34) cells, then cultured/expanded under optimal conditions for 2 weeks. Surgery to generate a long-gap sciatic nerve injury was performed in athymic nude mice, after which the mice were divided into exercise (E) and non-exercise (NE) groups. The mice were housed in standard individual cages, and voluntary exercise wheels were introduced to the cages of the E group one week after surgery. After 8 weeks, the human Sk-34 cells were actively engrafted, and showed differentiation into Schwann cells and perineurial cells, in both groups. The recovery in the number of axons and myelin in the conduit and downstream tibial nerve branches, and the lower hindlimb muscle mass and their tension output, was consistently higher by 15–25% in the E group. Moreover, a significantly higher innervation ratio of muscle spindles, reduced pathological muscle fiber area, and acceleration of blood vessel formation in the conduit were each observed in the E group. These results showed that the combined therapy of tube-bridging, Sk-34 cell transplantation, and voluntary exercise is a potentially practical approach for recovery following long-gap nerve injury.

## 1. Introduction

Serious losses in the vital functions of the somatic (motor and sensory) nervous system are induced by long-gap peripheral nerve transection injuries caused by mechanical trauma, such as penetration, crush, traction or laceration [1,2], and typically result in poor functional recovery [3,4]. Therefore, various methods, such as bridging the nerve gap with a tube of some kind, combined with stem cell transplantation, have been attempted to improve recovery, as alternatives to the current surgical standard, nerve autograft therapy. However, it is unlikely that a significantly better therapy, which goes well beyond the standard of care, has been established to date. With this background in mind, we have proposed and begun to develop a potential new therapy, which involves bridging the nerve gap with an acellular conduit combined with transplantation of skeletal muscle-derived stem cells (Sk-SCs) [5]. We previously demonstrated the high numerical and functional therapeutic capacity of this approach (2–3 fold higher capacity than nerve autograft therapy) using human Sk-SCs sorted as the CD34^+^/45^−^ fraction (Sk-34 cells) [6].

At the same time, the beneficial effects of exercise loading for the recovery of nerve injury have also been well documented. Treadmill exercise, performed during the first two weeks after peripheral nerve transection, was shown to enhance axon regeneration of the mouse common fibular nerves [7]. The same kind of treadmill exercise also enhanced motor axon regeneration, without increasing misdirected axonal regrowth, after transection and surgical repair of the mouse sciatic nerve [8]. Moreover, treadmill exercise-induced functional recovery following peripheral nerve repair has also been associated with increased levels of neurotrophic factors [9]. In contrast, although upslope treadmill exercise enhanced motor axon regeneration, it did not improve functional recovery following sciatic nerve transection and repair [10]. These results suggest that exercise may be an effective enhancer of the peripheral nerve repair process, however, considerable uncertainty remains. In particular, there is no direct evidence of an effect of post-injury exercise following treatment with tube-bridging and stem cell transplantation on severe long-gap nerve transections.

Thus, we examined the effect of voluntary wheel-exercise after nerve bridging with an acellular conduit associated with human Sk-34 cell transplantation, as an additional treatment for our proposed therapy. Wheel exercise was provided for the mice using individual free access wheel equipment. Preparation and transplantation of human Sk-34 cells, the generation of a long-gap (7 mm) sciatic nerve injury model, and bridging of the gap with an acellular conduit were performed in strict accordance with protocols described in a previous report [6,11]. Our results clearly indicate the positive effects of post-nerve injury exercise.

## 2. Experimental Section

### 2.1. Collection of Human Skeletal Muscle Samples

Three patients (aged 22 to 77 years, 2 males, 1 female) whose legs were amputated after unforeseen accidents contributed muscle tissue for this study. Study protocols were carried out according to The Code of Ethics of the World Medical Association (Declaration of Helsinki), and were approved by our institutional ethics committee (Tokai University School of Medicine: BC approval No. 12I-11). All patients gave written (signed) informed consent after being given a detailed explanation of the study’s aims and procedures. The consent procedures were also approved by our institutional ethics committees (see above). Muscle samples (5–10 g) were wrapped in gauze moistened with cold (4 °C) physiological saline immediately after removal, and were transferred to the laboratory for isolation of stem cells within 30 min.

### 2.2. Isolation of Skeletal Muscle-Derived Stem Cells (Sk-SCs), Cell Sorting, and Expansion

The human Sk-SCs were isolated using a previously described procedure for mouse and human muscles [5,6,11,12,13]. Briefly, muscle samples were weighed and washed several times with Dulbecco’s Modified Eagle’s medium (DMEM) containing 1% penicillin/streptomycin, and were cut into several pieces (5–7-mm thick and wide, and 40–50-mm long). Note that the muscles were never minced during the procedure. Muscle pieces were treated with 0.1% collagenase type IA (Sigma-Aldrich, St. Louis, MO, USA) in DMEM containing 7.5% fetal calf serum (FCS) with gentle agitation for 2 h at 37 °C. Extracted cells were filtered through 70-µm, 40-µm, and 20-µm nylon strainers in order to remove muscle fibers and other debris. Subsequently, cells were washed and resuspended in Iscove’s modified Dulbecco’s medium (IMDM) containing 10% FCS, yielding enzymatically extracted cells. Enzymatically extracted mixed cells were stored in liquid nitrogen with cell preservative solution (Cell Banker; Juji-field, Tokyo, Japan) until use, after pre-freezing at −80 °C using a bio freezing vessel (BICELL; Nihon Freezer Co., Ltd., Tokyo, Japan).

Sorting of enzymatically isolated cells and expansion culture was performed in strict accordance with a previous report [6,11]. Isolated cells were sorted using cell surface markers, with CD29 (BD Biosciences, San Jose, CA, USA), CD34 (RAM34, eBioscience, San Diego, CA, USA), and CD45 (30-F11, BioLegend, San Diego, CA, USA) antibodies using a FACSAria (Becton Dickinson Japan, Tokyo, Japan), to yield CD34^+^/45^−^ (Sk-34) and CD34^−^/45^−^/29^+^ (Sk-DN/29^+^) fractions. In the present study, only sorted Sk-34 cells were used after expansion culture in the optimal conditions described previously, because of the cell differentiation specificity to peripheral nerve support cells [6,11]. Cell passages were strictly limited to two times during expansion.

### 2.3. Recipient Animals, Voluntary Wheel Exercise and Cell Transplantation

Athymic nude mice (female, BALB/cA Jcl-*nu/nu*; CLEA Japan, Tokyo, Japan, aged 5–6 week, *n* = 12) were used as transplant recipients. The animals were housed in standard cages after the operation for one week. Then, the animals were divided into two different conditions, namely: (1) the non-exercise (NE group) housed in the same standard cages; and (2) the exercise (E group) housed in the standard cages attached with an activity wheel (diameter 140 mm, circumference 0.5 m, with magnetic rotation sensor, RW-15S, MELQUEST, Tokyo, Japan). Mice in the E group were given free access to an activity wheel from one week after surgery. All animals were provided food and water ad libitum, the room temperature was kept at 23 ± 1 °C, and a 12 h:12 h light-dark cycle was maintained throughout the experiment. During the recovery phase, the activity wheel rotation counter was checked every day at 17:00.

In order to determine the effect of voluntary exercise on the recovery from severe nerve injury, we used a completely transected nerve with long-gap model. Details of this model have been described previously [5]. The right sciatic nerve in all mice was transected with a 7 mm long, then bridged using an acellular conduit (12–15 mm long), and the length of gap was adjusted to 7–10 mm. The acellular conduit was made from a separated esophageal submucosal membrane collected from nude mice after 3 days of 70% ethanol dehydration, as we ordinarily stock [6]. The bridging conduit was injected with human Sk-34 cells (3 × 10^6^ cells/3 μL DMEM, per nerve). All operations were performed under inhalation anesthesia (Isoflurane; Abbot, Osaka, Japan), and body (rectal) temperature was maintained at 36 ± 1 °C with radiant heat throughout the surgical procedure. During surgery, analgesic nonnarcotic opioid (butorphanol tartrate; 0.1 mg/kg subcutaneous infusion, Meiji Seika Pharma, Tokyo, Japan) was administered, as needed. All experimental procedures were approved by the Tokai University School of Medicine Committee on Animal Care and Use (No. 153015). All methods were undertaken to minimize potential pain and distress, and no animals died unexpectedly during the study.

### 2.4. Functional Assessment of Downstream Muscles

As the prominent functional recovery markers for the long-gap sciatic nerve transection, tetanic tension outputs of the downstream muscles, the lower hindlimb plantar flexor muscles of nude mice were measured in both the left (non-operated control side) and right (operated side) legs, and compared between E and NE groups. Measurements were performed in situ under inhalation anesthesia (Isoflurane; Abbot, Osaka, Japan), and body (rectal) temperature was maintained at 36 ± 1 °C with radiant heat throughout the measurement. Tension was measured separately by solo plantaris (PLA) and combined soleus (SOL) + gastrocnemius (GAS) muscles, and then added. The distal tendons of reference muscles and sciatic nerves (about 10 mm) on both sides were carefully exposed, and tissues were coated with mineral oil to prevent them from drying and to minimize electric noise interference. The details for setting the instrument and the method of the tension measurement have been described previously [5,6]. Tetanic tension output was considered to represent total recovery of nerve-muscle units, and the recovery ratio was determined based on the contralateral side.

### 2.5. Immunohistochemical Analysis

**Figure 1:**
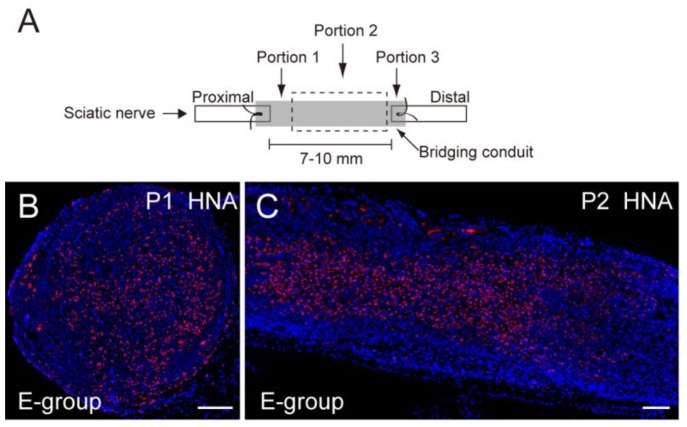

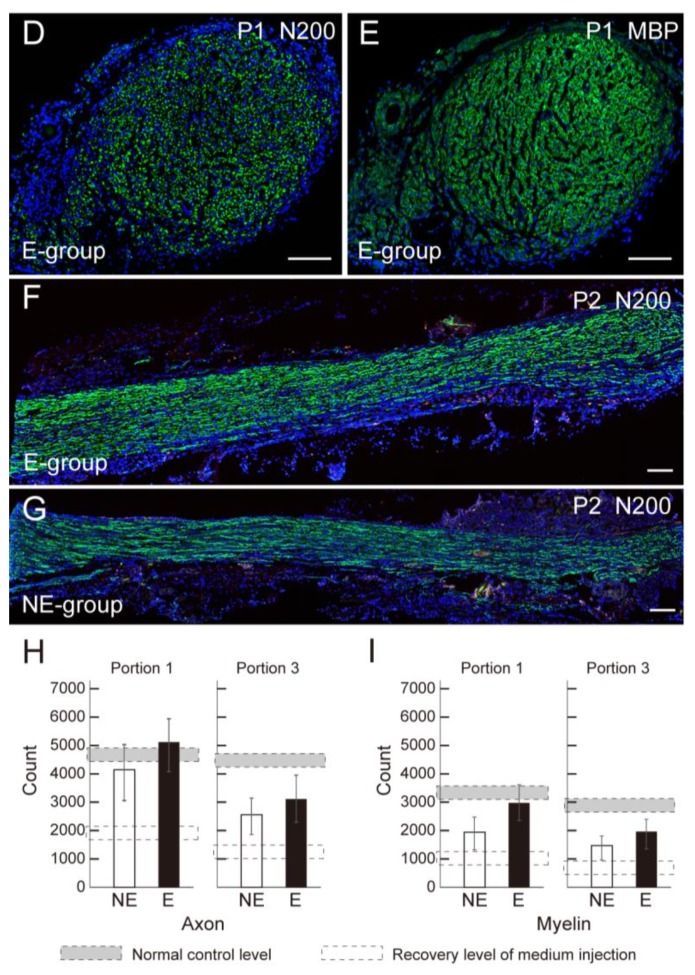
Extraction of histological sections and comparison of the number of axons and myelin in the conduit. (**A**) Histological sections were obtained from three portions; Portions 1 and 3 are cross-sections, and Portion 2 is a longitudinal section; (**B**,**C**) Typical engraftment of human Sk-34 cells stained with HNA (human nuclear antigen, red) in cross- (**B**) and longitudinal (**C**) sections; (**D**,**E**) Typical staining of axons (**D**, N200, green), and typical staining of myelin (**E**, MBP; myelin basic protein, green) E-group; (**F**,**G**) Similarly, typical staining of axons (green) in longitudinal sections (Portion 2) both in E-group (**F**) and NE-group (**G**); Axonal regeneration is apparent through the conduit in both groups. Blue staining is nuclear staining of 4′,6-diamidino-2-phenylindole (DAPI). P1 and P2 correspond to Portions 1 and 2, respectively. Bars in B-E = 100 μm; (**H**) Axon numerical recovery in Portions 1 and 3; (**I**) Myelin numerical recovery in Portions 1 and 3. The E group consistently showed greater recovery of axon and myelin in the conduit than the NE group. NE = non-exercise group, E = exercise group. Gray dotted square shows the mean number of axons in the same portion of a normal control sciatic nerve based on our pooled data. An open dotted square shows the recovery level of the case of conduit + non-cell media injection derived from our pooled data.

At 8 weeks after transplantation, recipient nude mice were given an overdose of pentobarbital (60 mg/kg, i.p.), and the animal was exsanguinated. Then, the sciatic nerve in each side was removed and fixed overnight in 4% paraformaldehyde/0.1 M phosphate buffer (4% PFA/PB), washed with graded sucrose (0–25%)/0.01 M phosphate-buffered saline (PBS) series, embedded in optimum-compound (O.C.T compound; Tissue-Tek, Sakura Finetechnical Co., Ltd., Tokyo, Japan) and then frozen at −80 °C, and stored until sectioned. Similarly, plantar flexor muscles (PLA, SOL, and GAS) were freshly removed and quickly frozen in isopentane pre-cooled with liquid nitrogen and stored at −80 °C until sectioned. Subsequently, in order to examine the bridged conduit, several 7 µm cross- and longitudinal sections were obtained from three portions, as shown in Figure 1A. Portion 1 was a cross-section showing the proximal portion of the conduit, portion 2 was a longitudinal section showing the midportion of the conduit, and portion 3 was a cross-sectional profile of the distal portion of the conduit. Engrafted human cells were detected by anti-human nuclear antigen (HNA, 1:100, 4 °C overnight; clone 235-1, Cy3 conjugate; Millipore, Temecula, CA, USA). The localization of the nerve fibers (axons) was determined by rabbit polyclonal anti-neurofilament 200 (N-200, 1:1000, room temperature for 1 h; Sigma, Saint Louis, MO, USA). The myelin formation was detected by rabbit polyclonal anti-myelin basic protein (MBP; 1:200, room temperature for 2 h; Millipore, Billerica, MA, USA). The total distribution of blood vessels was determined using rat anti-mouse CD31 monoclonal antibody (1:500, 4 °C overnight; BD Pharmingen, San Diego, CA, USA), and/or rabbit polyclonal anti-human CD31 (1:200, room temperature for 2 h; Abcam, Cambridge, UK). Immature Schwann cells were detected using rabbit anti-p75 polyclonal antibody (1:400, 4 °C overnight; CST, Boston, MA, USA) or mouse monoclonal antibody (1:100, 4 °C overnight; Abcam, Tokyo, Japan), and rabbit anti-glucose transporter 1 (GLUT-1, 1:100, room temperature for 1 h; Diagnostic BioSystems, Pleasanton, CA, USA) was used for identification of the perineurium. Muscle fibers were confirmed with rabbit polyclonal anti-fast myosin skeletal muscle heavy chain (1:300, room temperature for 1 h; Abcam, Tokyo, Japan). Reactions were visualized using Alexa Fluor-488, -594, -647-conjugated goat anti-rabbit, anti-rat antibodies (1:500, room temperature for 2 h; Molecular Probes, Eugene, OR, USA), respectively. Nuclei were counter-stained with 4′,6-diamidino-2-phenylindole (DAPI). Histological photographs were taken with a fluorescence multi focal projection system using Stereo Investigator (mbf Bioscience, MicroBrightField. Inc., Williston, VT, USA) and fluorescence microscopy (Olympus BX61 with U-HGLGPS and BX-UCB, Tokyo, Japan). Histological analyses were performed on all samples.

### 2.6. Characterization of Downstream Plantar Flexor Muscles and Muscle Spindles, and Associated Tibial Nerves

In order to further examine the recovery of the downstream nerve-muscles, histological sections were obtained from combined planter flexor muscles (GAS, PLA, SOL). Then, axon staining with N-200, laminin, CD31 and fast-myosin heavy chain staining were performed. Characteristics of muscle fibers, tibial nerve branches, and the muscle spindles in the planter flexor muscles were analyzed and quantified for comparison between E and NE groups. Similarly, myosin ATPase staining (preincubation at pH 4.2~4.3 and specifically stained for Type-I fiber) was also performed in order to examine the location of the GAS, PLA, and SOL muscles. The number of axons and myelin sheaths in the tibial nerve branches, innervation to muscle spindle, and pathological muscle fiber areas (such as denervation induced muscle fiber atrophy characterized by extremely small fiber diameters and with central nuclear) were identified and quantified as the percentage of the total cross-sectional area.

### 2.7. Quantitative Analysis

Regeneration of axons, myelin, and blood vessels in whole cross-sections of the bridging conduit, as well as the characteristics in the downstream muscle was determined by the number of positive reactions to anti-N200, -MBP, and -CD31, respectively. Reacted cells/tissues were counted using a Stereo-investigator (MBF Bioscience, MicroBrightField, Inc., Williston, VT, USA). The analysis was performed on 4–5 sections per sample, and values were averaged. Values are expressed as means ± SE. Differences in morphological and functional data between groups were evaluated by the student’s *T*-test, and the level of significance accepted was set at *p* < 0.05.

## 3. Results

### 3.1. Recovery of the Number of Axon and Myelin in the Nerve Bridged Conduit

Axon and myelin recovery was evaluated at the proximal site (Portion 1) and distal site (Portion 3) in the conduit (Figure 1A) after 8 weeks of transplantation. Figure 1B,C shows typical engraftment of the transplanted human Sk-34 cells. The Sk-34 cells were evenly distributed in the conduit transversely (Portions 1 and 3) and longitudinally (Portion 2). Similarly, regenerated axon and myelin at portion 1 is shown in Figure 1D,E. Axonal regeneration in the longitudinal sections of both the E- and NE-group are also shown in Figure 1F,G. A similar trend, the axons running through the conduit, was evident in both groups. Then, these regenerated axons and myelin were counted and compared (Figure 1H,I). Axonal recovery of the E group reached the normal control level in Portion 1 and about 70% of normal in Portion 3 (Figure 1H). Similarly, myelin recovery in the E group showed almost 90% of the normal control level in Portion 1 and about 70% in Portion 3 (Figure 1I). On the other hand, the NE group showed consistently lower values than the E group, although these differences were not statistically significant. Note that both the present E and NE groups clearly showed significantly greater recovery than the conduit + medium injection level, based on our pooled data (see Figure 1H,I, and ref [6]).

### 3.2. Recovery of Downstream Plantar Flexor Muscle Mass and Tension Output, and Individual Exercise Score during the Recovery Period

**Figure 2:**
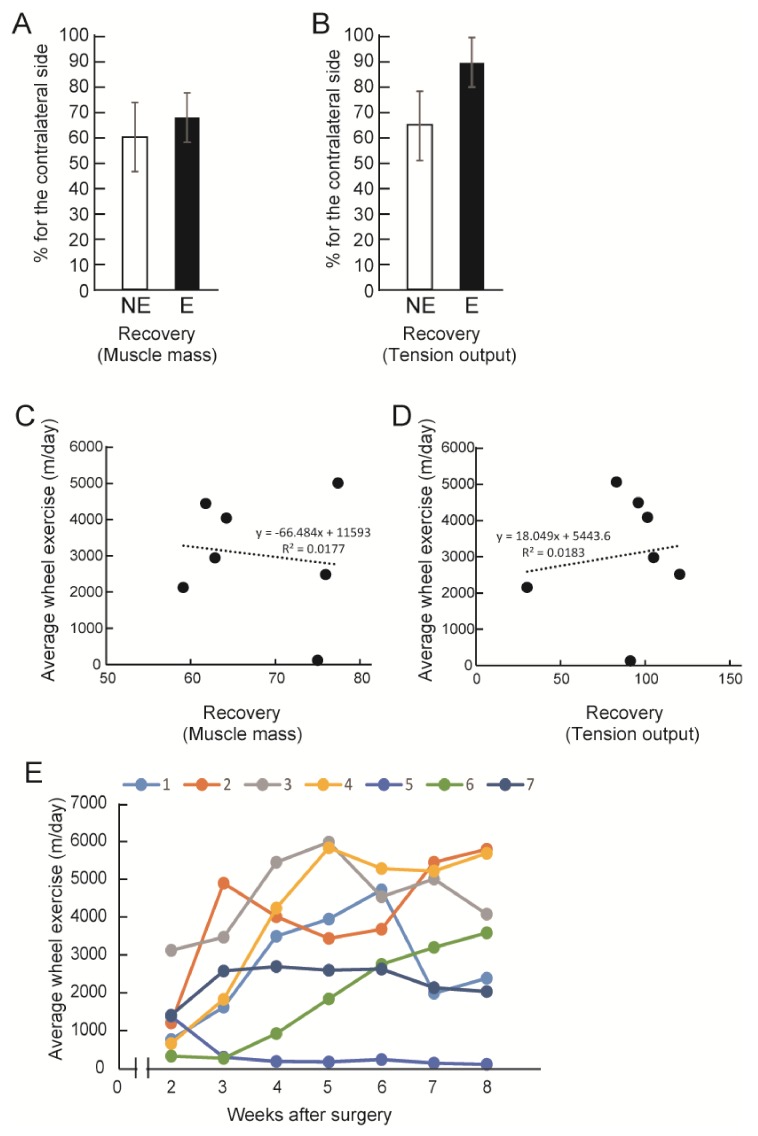
Recovery of downstream muscle mass and tension output, and individual exercise statistics. (**A**) Recovery of total muscle mass (gastrocnemius (GAS) + plantaris (PLA) + combined soleus (SOL)); (**B**) Recovery of tension output of the three muscles. Both markers are calculated based on the contralateral side. The E group consistently shows better recovery than the NE group; (**C**) Relationship between average wheel exercise (m/day) and muscle mass recovery; (**D**) Relationship between average wheel exercise (m/day) and muscle tension recovery; (**C**,**D**) shows individual plots, and there are no correlations; (**E**) Individual shift of exercise volume during recovery term.

The measurement of final body mass, the downstream muscle mass (GAS + PLA + SOL) and their tension output are summarized in Table 1. When the latter two parameters were compared as % recovery to the values of the contralateral side, the E group consistently showed higher values in both parameters, although they were not statistically significant (Figure 2A, B). In the individual observation of the E group, no significant relationship was found between the amount of exercise performed and the recovery of muscle mass and/or tension output (Figure 2C, D). For the individual time-dependent changes of voluntary wheel exercise, the E group showed individual differences, but in general we observed a trend of increasing activity over time (Figure 2E).

### 3.3. Axon Counts in the Downstream Tibial Nerve Branches

**Figure 3:**
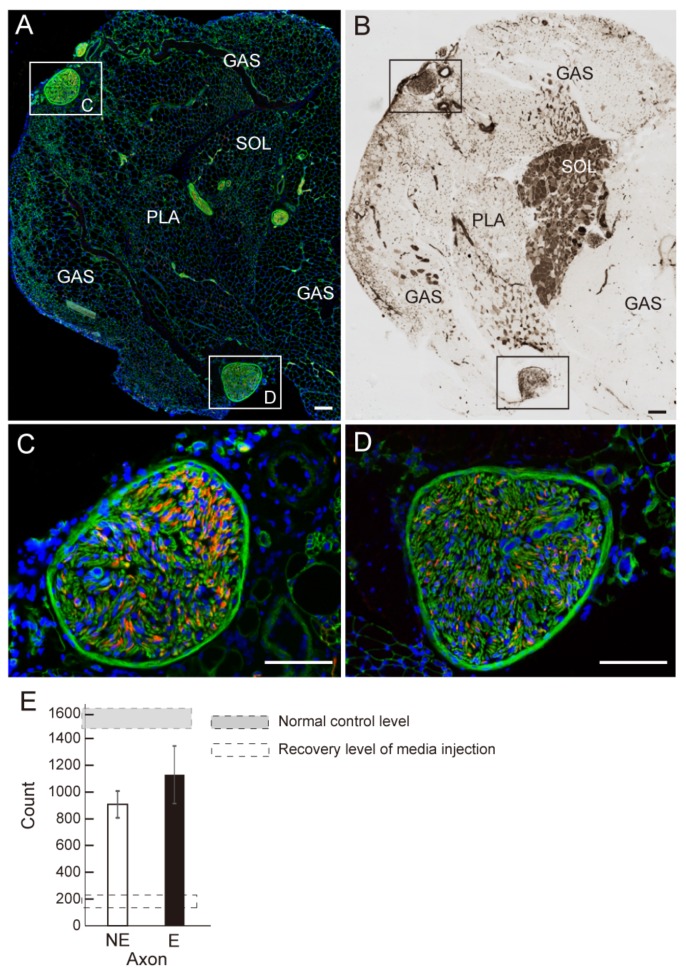
Detection and analysis of tibial nerve branches in the downstream muscles of E-group. (**A**) A typical histological section of the NE group showing the combined heads of gastrocnemius (GAS), planters (PLA) and Soleus (SOL) muscles, stained with N200 (red) and laminin (green); (**B**) ATPase (pH 4.3) staining of the same histological section. Location of the three muscles (GAS, PLA and SOL) is clear; (**C**,**D**) Panels (**C**,**D**) show higher magnification of tibial nerve branches derived from the portions in the squares in panel (**A**,**B**) stained with N200 (red) and laminin (green). Blue staining in (**A**,**C**,**D**) is nuclear staining of DAPI. Bars in A–D = 100 μm; (**E**) Comparison of the number of axons in the tibial nerve branches. In this analysis, the E group showed higher recovery than NE. However, the morphological characteristics of NE and E group were wholly similar. Gray dotted square shows the mean number of axons in the same portion of a normal control animals based on our pooled data. Open dotted square shows the recovery level in for conduit + non-cell media injection derived from our pooled data.

The number of axons of the tibial nerve branches that were apparent in the plantar flexor muscles was counted (Figure 3). The typical location of tibial nerve branches, as shown in Figure 3A,B, associate with the situation of plantar flexor muscles (GAS, PLA, SOL). These two branches include N200^+^ axons (Figure 3C,D). When these numbers are compared as the recovery ratio against the normal control levels, the E group showed about 73% recovery, with about 60% observed in the NE group (however, this difference is not statistically significant). Note that both groups showed significantly greater recovery than the medium injected case (our pooled data).

### 3.4. Characteristics of Downstream Muscle Spindle and Fibers

**Figure 4:**
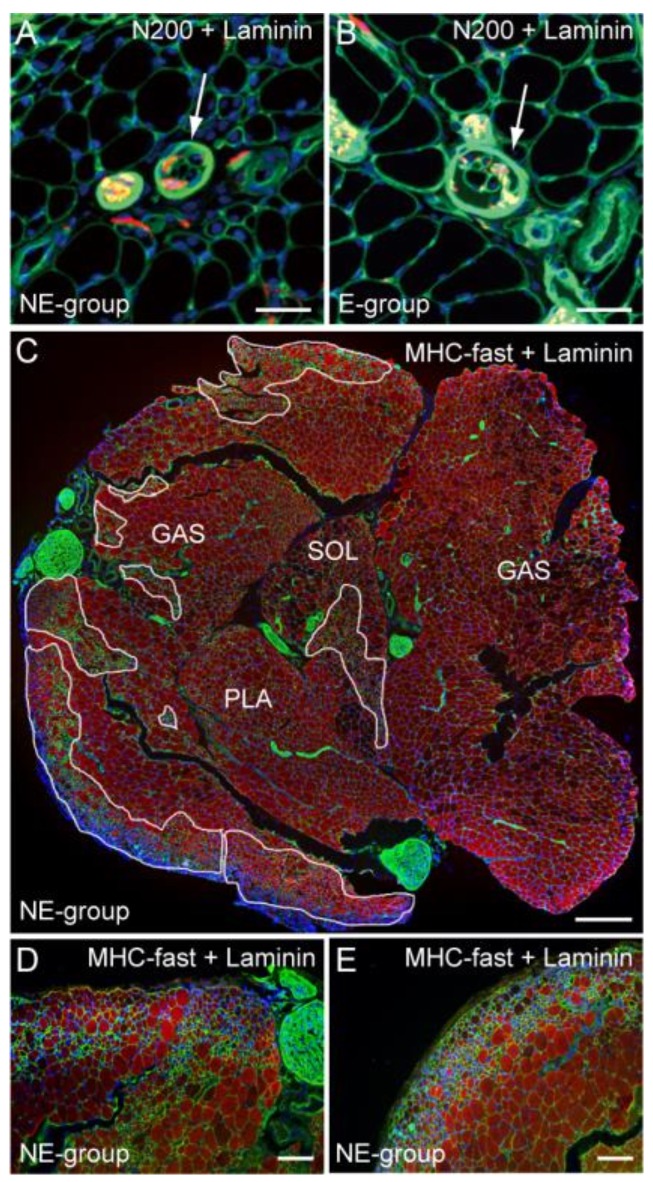
Detection of muscle spindles and pathological muscle fiber area. Typical muscle spindle in the NE (**A**, arrow) and E (**B**, arrow) group stained with N200 (red) and laminin (green). Both spindles are innervated, but irregular fiber diameters and central nuclei are observed in the NE group; (**C**) Detail of the pathological muscle fiber area in the whole muscle cross-section (GAS, PLA, SOL) stained with anti-myosin heavy chain (MHC) fast type (red) and laminin (green). These enclosed areas account for 17% as pathological area. A higher magnification of the typical pathological muscle fiber features is shown in (**D**,**E**). Severe irregular fiber diameters are evident. Blue staining in each panel is the nuclear staining of DAPI. Bars in (**A**,**B**) = 50 μm, (**C**) = 200 μm, and (**D**,**E**) = 100 μm.

Morphological aspects of downstream muscle spindles and fibers are shown in Figure 4. As the marker of sensory system recovery, innervation of muscle spindle was examined. Typical muscle spindles of the NE and E group are shown in Figure 4A,B. Both spindles included axons (arrows in Figure 4A,B), thus, they were considered viable and functional. However, irregular fiber diameters are evident only in the NE group (Figure 4A). Detection of the pathological muscle fiber area on the whole cross-section of the three muscles combined, stained with MHC-fast type and laminin, is shown in Figure 4C. The pathological fiber area was enclosed by white lines, and these accounted for 17% of the total area. Detail of the pathological fiber area is shown in Figure 4D,E. Extremely small fibers, affected by denervation, were aggregated.

**Figure 5:**
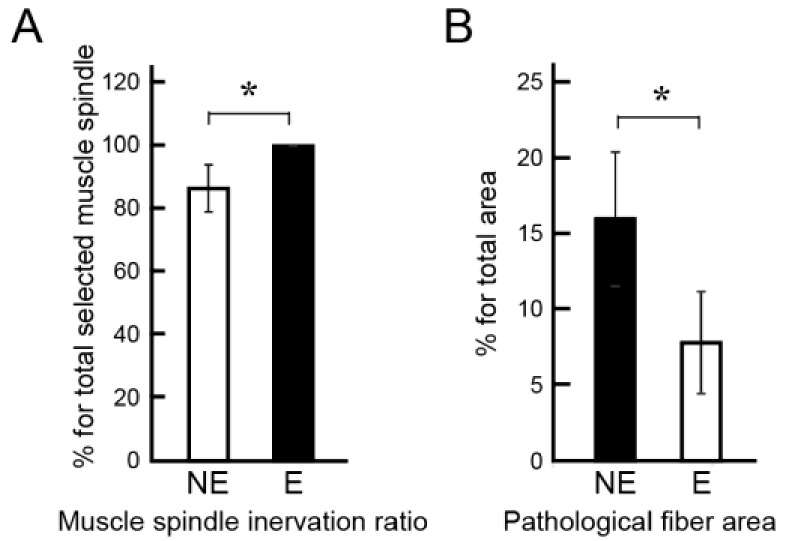
Comparison of the innervation of muscle spindle and pathological fiber area. (**A**) Muscle spindle innervation ratio determined by 23 spindles. The E group showed 100% innervation, which is significantly higher than NE group (87%); (**B**) Existence of pathological muscle fiber area account for a percentage of total area of cross-section. The E group showed significantly lower existence of pathological fibers. * *p* < 0.05.

In order to further examine nerve recovery by the combined therapy of tube bridging and Sk-34 cell transplantation, the above characteristics were quantified as the innervation ratio of muscle spindle, with the inclusion of the pathological fiber area (Figure 5). The results showed that significantly higher innervation of the muscle spindle was observed in the E group (Figure 5A), as well as significantly lower inclusion of pathological fiber area (Figure 5B).

### 3.5. Differentiation of Transplanted Human Sk-34 Cells and Blood Vessel Formation in the Conduit

**Figure 6:**
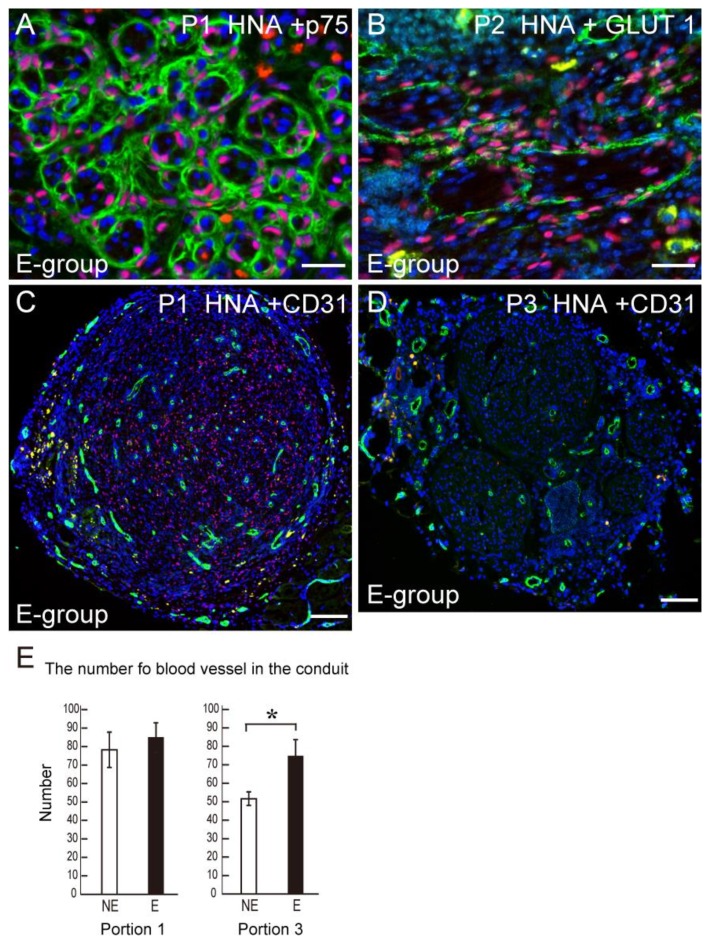
Differentiation of engrafted human Sk-34 cells into Schwann cells and perineurial cells, and the number of blood vessels in the bridging conduit. (**A**) Transplanted human Sk-34 cells (pink nuclei) expressed a newly formed Schwann cell marker p75 (green); (**B**) Transplanted human Sk-34 cells (pink nuclei) also formed new perineurium (green) showing differentiation into perineurial cells; (**C**) Typical staining of CD31 (green) as evidence of the number of blood vessels in the conduit. Blue staining in each panel is the nuclear staining of DAPI. P1 = position 1, P2 = position 2. Bars in A and B = 50 μm, C = 100 μm; (**D**) Statistical comparison of the formation of blood vessels in the conduit. There is no difference in the proximal Portion 1 between NE and E group, but a significantly higher value in (**E**) was observed in the distal end Portion 3. * *p* < 0.05.

Transplanted human Sk-34 cells differentiated into anti-p75^+^ Schwann cells (Figure 6A). In addition, Sk-34 cells also formed GLUT-1^+^ perineurium (Figure 6B) showing a differentiation into perineurial cells. Typical blood vessel formation detected by CD31 is shown in Figure 6C. At the conduit portion 1, transplanted human Sk-34 cells (HNA^+^ cells) and blood vessels (CD31^+^) uniformly distributed in the conduit (Figure 6C). When the number of CD31^+^ blood vessels were counted and compared, there was no difference at the proximal portion 1 of conduit, however, a significant difference was observed at the distal portion 3 (Figure 6D).

## 4. Discussion

Our proposed method, tube bridging with human Sk-34 cell transplantation, has been shown to have a 2–3 fold higher therapeutic capacity for the long-gap injury than the current standard of care, autologous nerve graft, numerically and functionally without additional exercise treatment [6]. In this regard, the present NE group also showed sufficiently high recovery, thus, there is a little room for improvement by some means. However, the present study sought additional methods to be obtained for further evidence of nerve recovery, such as numerical (axon and myelin counts), motor and sensory functions, and shortening of the recovery period. In these regards, the present voluntary wheel exercise (E group) consistently showed an enhanced trend of these recoveries. We believe that a consistent trend in a similar direction may show important details that are not statistically significant.

The number of axons/myelin in the tube, the contractile function, and the muscle mass of the downstream muscles were all higher in the E group (Figure 1F,G). This demonstrates that the re-connection of the gap-transected nerve fibers was accelerated by exercise, and this was further reflected in the recovery of muscle mass and the contractile function of downstream muscles (Figure 2A,B). This functional recovery was also supported by the recovery of axons in the tibial nerve branches (Figure 3E). These are not unexpected responses. However, in the analysis of individual mice in the E group, no significant relationship was found between the amount of exercise performed and the recovery of muscle mass and/or tension output (Figure 2C,D). This means that the amount of exercise did not directly affect the recovery of the nerve injury. Instead, this may be a matter of whether the exercise was performed or not during the recovery term. Therefore, there is still little evidence of an effect of post-injury exercise on axon and/or functional regeneration in the peripheral nervous system. It was suggested that the exercise quality and/or intensity may be more effective factors for nerve regeneration [7,10], instead of exercise volume. Thus, exercise quality may be a more dominant factor than exercise volume. The control of exercise quality may be easier in the case of human patients, because of the variations of rehabilitation technology [14].

With respect to the downstream muscles, the innervation ratio of muscle spindle was also significantly higher in the E group (Figure 5A). We used this parameter as the indicator of sensory nerve recovery. In this regard, the E group showed 100% recovery, thus, voluntary exercise contributed to enhance the re-connection of afferent sensory nerve fibers. Similarly, the pathological muscle fiber area, mainly composed of extremely small diameter fibers, was also significantly lower in the E group (Figure 5B). These extremely small fibers are considered fibers which have not been re-established by motor nerve innervation even at 8 weeks after surgery, and thus, can also be considered proof of the recovery ratio of the motor unit according to the re-connection of efferent nerve fibers. In this regard, this is further evidence that the free wheel-exercise contributes to the recovery of motor unit and/or re-connection of efferent motor nerve fibers.

Paracrine/autocrine effects on nerve growth and/or neurotrophic factors from the growth cones have been suggested as reasons to explain the mechanism of exercise induced enhancement of the re-connection of gap-transected motor and sensory nerves [9]. BDNF is a prominent candidate to link exercise and the enhanced axonal regeneration [15,16]. The transcription factor Sox11 is also an underlying candidate for the mechanism of exercise-induced facilitation of sensory nerve regeneration [17,18,19]. Secreted BDNF binds to trkB (tropomyosin-related kinase B) receptors [20] on the same and adjacent axons and promotes outgrowth. In the previous study, co-transplanted Sk-34 cells also exerted paracrine effects for the nerve regeneration process [6,21], but the expression of BDNF and Sox11 was not confirmed at the protein level, although the mRNA was detected. Thus, additional effects of BDNF and Sox11 on axonal outgrowth may be expected in future analyses of the E group in the present study.

Similar to the exercise effect above, electrical stimulation facilitated nerve regeneration has been reported as an alternative strategy [22,23,24]. The combined effects of electrical stimulation and exercise was also reported to increase peripheral nerve axonal regeneration [25]. With respect to clinical applications, electrical stimulation is likely to be an easier approach than exercise, particularly in older patients. Therefore, the combined use of nerve bridging, Sk-34 transplantation and electrical stimulation is an additional choice. However, the effects on downstream muscle function are likely to be much higher with exercise loading [25]. Therefore, multiple combinations of the technologies described above should be considered on a case by case basis in order to obtain the best results.

## 5. Conclusions

In conclusion, the voluntary free wheel-exercise following the long-gap nerve transection therapy using tube bridging with human Sk-34 transplantation, potentially induced a consistent trend of acceleration in the quantitative recovery, including the number of re-connected axons in the tube and downstream muscle contractions, in addition to significant recovery of re-innervation in the muscle spindle (sensory) and motor units, and was also associated with significantly enhanced formation of blood vessels in the tube. Therefore, the combination of tube bridging, transplantation of Sk-34 cells and voluntary exercise may represent an improved method for treating peripheral nervous system injury. However, it is also likely that the exercise regimens/programs need to be closely monitored and controlled to support better regeneration and recovery.

## Figures and Tables

**Table 1:** Final Body mass, and downstream muscle mass and tension output (8 weeks after operation).

	Body Mass (g)	Muscle Mass (mg) *		Tension Output (N 1 × 10^2^) *	
		Op-Side	Con-Side	Op-Side	Con-Side
E group	24.3 ± 0.6	84.5 ± 7.5	133.0 ± 4.9	75.8 ± 11.2	81.2 ± 4.7
NE group	23.2 ± 0.4	74.5 ± 9.5	132.5 ± 3.9	57.4 ± 8.3	94.2 ± 9.6

* Muscle mass and Tension output = total of gastrocnemius, soleus and plantaris muscles. Op-side = operated-side, Con-side = control-side.
